# The novel BMI-1 inhibitor PTC596 downregulates MCL-1 and induces p53-independent mitochondrial apoptosis in acute myeloid leukemia progenitor cells

**DOI:** 10.1038/bcj.2017.8

**Published:** 2017-02-17

**Authors:** Y Nishida, A Maeda, M J Kim, L Cao, Y Kubota, J Ishizawa, A AlRawi, Y Kato, A Iwama, M Fujisawa, K Matsue, M Weetall, M Dumble, M Andreeff, T W Davis, A Branstrom, S Kimura, K Kojima

**Affiliations:** 1 Division of Hematology, Respiratory Medicine and Oncology, Department of Internal Medicine, Saga University, Saga, Japan; 2 PTC Therapeutics, South Plainfield, NJ, USA; 3 Section of Molecular Hematology and Therapy, Department of Leukemia, The University of Texas MD Anderson Cancer Center, Houston, TX, USA; 4 Department of Cellular and Molecular Medicine, Graduate School of Medicine, Chiba University, Chiba, Japan; 5 Division of Hematology/Oncology, Department of Medicine, Kameda Medical Center, Kamogawa, Japan; 6 Bristol-Myers Squibb, Princeton, NJ, USA; 7 PMV Pharmaceuticals Inc., Cranbury, NJ, USA

## Abstract

Disease recurrence is the major problem in the treatment of acute myeloid leukemia (AML). Relapse is driven by leukemia stem cells, a chemoresistant subpopulation capable of re-establishing disease. Patients with p53 mutant AML are at an extremely high risk of relapse. B-cell-specific Moloney murine leukemia virus integration site 1 (BMI-1) is required for the self-renewal and maintenance of AML stem cells. Here we studied the effects of a novel small molecule inhibitor of BMI-1, PTC596, in AML cells. Treatment with PTC596 reduced MCL-1 expression and triggered several molecular events consistent with induction of mitochondrial apoptosis: loss of mitochondrial membrane potential, BAX conformational change, caspase-3 cleavage and phosphatidylserine externalization. PTC596 induced apoptosis in a p53-independent manner. PTC596 induced apoptosis along with the reduction of MCL-1 and phosphorylated AKT in patient-derived CD34^+^CD38^low/−^ stem/progenitor cells. Mouse xenograft models demonstrated *in vivo* anti-leukemia activity of PTC596, which inhibited leukemia cell growth *in vivo* while sparing normal hematopoietic cells. Our results indicate that PTC596 deserves further evaluation in clinical trials for refractory or relapsed AML patients, especially for those with unfavorable complex karyotype or therapy-related AML that are frequently associated with p53 mutations.

## Introduction

Acute myeloid leukemia (AML) is a clonal hematopoietic disorder resulting from genetic alterations in normal hematopoietic stem cells. AML is a disease of older adults, with a median age at diagnosis of ~70 years.^1^ The outcome of AML remains unsatisfactory, especially in elderly patients who often suffer from unfavorable complex karyotype or therapy-related AML and are generally ineligible for allogeneic stem cell transplantation.^2, 3^ The 5-year survival rate is only 3–8% for patients over age 60 and 30–40% for younger patients.^1, 2, 3^ Recent studies have shown that mutations of the tumor suppressor p53 occur frequently in patients with complex karyotype (60–80%) and therapy-related AML (30%).^4, 5^ More importantly, p53 mutations are an independent predictor of very poor outcome (survival at 3 years of ~0%).^6^ Therefore, the development of novel agents that induce AML cell death through p53-independent mechanisms is critical.^7^

Leukemia stem cells are constitutionally resistant to chemotherapeutic agents and thus represent a major obstacle in establishing a successful cure for AML. Studies have demonstrated that B-cell-specific Moloney murine leukemia virus integration site 1 (BMI-1) has vital roles in the self-renewal and maintenance of AML stem cells,^8, 9^ as well as normal hematopoietic stem cells.^8, 10, 11, 12^ BMI-1 represses the transcription of a range of target genes, including *CDKN2A* (which encodes INK4A and ARF) and *HOX* cluster genes.^13, 14^ BMI-1 has been reported to be overexpressed in AML,^15, 16^ and the TCGA data sets in the cBioPortal for Cancer Genomics (http://www.cbioportal.org/) show that AML cells express the highest levels of BMI-1 among 30 cancer cell types.^17, 18^ Importantly, higher levels of BMI-1 have been associated with more aggressive disease and poorer outcome for AML patients.^15, 16, 19^ As BMI-1 knockdown has been found to impair self-renewal and induce apoptosis in AML stem cells,^20^ we hypothesized that a therapeutic strategy targeting BMI-1 could eradicate chemoresistant AML stem cells. BMI-1 and p53 function in the BMI-1–ARF–MDM2–p53 signaling pathway,^7^ and reduced BMI-1 expression could potentially trigger p53-mediated apoptosis. The relative contribution of p53-dependent apoptosis to total apoptosis induced by BMI-1 knockdown, however, has not been clarified in AML.^20^

Recently, researchers have reported the preclinical utility of the first BMI-1 inhibitor PTC-209 in various cancers including colorectal cancer,^21^ biliary tract cancer,^22^ breast cancer,^23^ ovarian cancer,^24^ prostate cancer,^25^ chronic myeloid leukemia,^26^ multiple myeloma^27^ and AML.^19^ In mouse models with patient-derived colorectal cancer, PTC-209 potently suppressed tumor growth and eradicated cancer-initiating cells.^21^ We reported that PTC-209 induces apoptosis in AML patient-derived CD34^+^CD38^low/−^ stem/progenitor cells.^19^ Unfortunately, PTC-209 is not suitable for use in humans because of its limited potency and poor pharmacokinetic properties, and it has not entered clinical trials. PTC596 is another small molecule that was identified in a high-throughput small molecule library screen as potent repressor of BMI-1 in tumor cells, with a favorable safety profile.^28^ PTC596 has entered a Phase 1 clinical trial in patients with advanced solid tumors (NCT02404480). Early mechanism-of-action studies suggested that PTC596 induces binding of CDK1 to BMI-1 and CDK1-mediated phosphorylation of BMI-1 at two novel N-terminal sites, leading to degradation of BMI-1.^28^ In this study, we investigated preclinical *in vitro* and *in vivo* activities of PTC596 and the mechanisms of action in AML, having special attention to p53-dependency in inducing apoptosis.

## Materials and methods

### PTC596 synthesis and other reagents

A high-throughput small molecule library screen at PTC Therapeutics, South Plainfield, NJ, USA, identified candidates that reduce endogenous BMI-1 levels in tumor cells; after chemical optimization through structure-activity-relationships followed by *in vitro* and *in vivo* studies, PTC596 was discovered.^28^ Cycloheximide, MG132 and Z-VAD-FMK were purchased from Axxora (San Diego, CA, USA). The selective small molecule antagonist of MDM2, Nutlin-3a, was purchased from Cayman Chemical Company (Ann Arbor, MI, USA).

### Cell culture

Cell lines were cultured in RPMI 1640 medium containing 10% heat-inactivated fetal bovine serum. MOLM-13, MOLM-14, OCI-AML3 and MV4-11 cells express wild-type p53, whereas U-937 (p.G187_splice), HL-60 (p.M1_*394del) and K562 (p.Q136fs*13) cells have p53 mutations. Cell lines were harvested in log-phase growth, seeded at a density of 1–2 × 10^5^ cell/ml and exposed to compounds. Cell viability was evaluated by triplicate counts of Trypan blue dye-excluding cells. Patient samples were analyzed under the protocol approved by the institutional review board at Saga University (2014-10-5). Heparinized peripheral blood or bone marrow samples were obtained from AML patients after informed consent, according to institutional guidelines per the Declaration of Helsinki. Primary AML cells were seeded at density of 5 × 10^5^ to 1 × 10^6^ cells/ml cultured in the StemSpan SFEM medium with StemSpan CC100 cytokine cocktail (Stem Cell Technologies, Vancouver, Canada), and were exposed to PTC596. PTC596 was provided by PTC Therapeutics, South Plainfield, NJ, USA.

### Transfection of BMI-1 siRNA

MOLM-13 and U-937 cells were transfected with small interfering RNA (siRNA) oligonucleotides with the Amaxa Nucleofector 2b device, using the Nucleofector Kit C (program X-001 for MOLM-13 cells and W-001 for U-937 cells; Lonza, Basel, Switzerland). Cells were transfected with negative control siRNA or with BMI-1 siRNA (HSS101040; Life Technologies, Carlsbad, CA, USA).

### Stable transduction of *BMI-1* cDNA

Stable MOLM-13 and K562 cell lines overexpressing BMI-1 were established as described previously.^9, 12^

### Flow cytometric analysis

For apoptosis analysis, flow cytometric determination of annexin V binding, mitochondrial membrane potential loss (Δψ_m_), conformational change in BAX and caspase-3 cleavage were carried out.^29^ For gating stem/progenitor population (CD34^+^CD38^low/−^ cells) in primary AML samples, anti-human CD34 and CD38 antibodies (BD Biosciences, San Jose, CA, USA) were used. Cell cycle distribution was analyzed by the Click-iT EdU incorporation kit (Life Technologies). Staining of intracellular BMI-1 and p53 was performed as described previously.^19, 30^ Expression level was measured as the mean fluorescence intensity ratio (MFIR) calculated by the formula: MFIR=(MFI for specific antibody)/(MFI for isotype control).

### CyTOF

Surface and intracellular staining and single-cell CyTOF (time-of-flight mass cytometry) analysis were performed as described previously.^31^ Data were saved in FCS3.0 format and analyzed by FlowJo software (version 10.1 for MacOS X; FlowJo, LLC, Ashland, OR, USA).

### Western blot analysis

Western blot analysis was performed as previously described.^30^ Antibodies against BMI-1, Histone H2A, Cyclin B1, CDK1, phosphorylated CDK1, phosphorylated CDK2, Securin and β-ACTIN were purchased from Cell Signaling Technology (Danvers, MA, USA); ubiquitinated histone H2A from EMD Millipore (Darmstadt, Germany); CDK2 from Santa Cruz Biotechnology (Santa Cruz, CA, USA); BCL-2 from Dako (Glostrup, Denmark); BCL-X_L_, MCL-1 and BAX from BD Biosciences; and conformation-specific BAX from Trevigen (Gaithersburg, MD, USA).

### Quantitative real-time PCR

The mRNA expression levels were quantified using TaqMan gene expression assays (*BMI1*, Hs00995536_m1; *MCL1*, Hs01050896_m1; *GAPDH*, Hs99999905_m1; Applied Biosystems, Foster City, CA, USA).

### *TP53* mutation analysis

Mutation analysis of *TP53* was performed as previously described.^32^

### Animal experiments

Animal studies were conducted in accordance with the guidelines approved by the Institutional Animal Care and Use Committees at the Saga University and Rutgers University. Three xenograft models were utilized. In the first model, 1 × 10^6^ MOLM-13 cells were injected intravenously into non-obese diabetic-severe combined immunodeficiency (NOD-SCID)/IL2Rγ-KO (NSG) mice (Jackson Laboratory, Bar Harbor, ME, USA). One day after injection (day 1), mice were randomized into two groups and treated with vehicle (0.5% hydroxypropyl methylcellulose and 0.1% Tween 80 in distilled water) or PTC596 (5 mg/kg) by oral gavage every 3 days (12 mice/group). On day 13, circulating leukemia cells were detected by flow cytometry using human- and mouse-specific anti-CD45 antibodies (BD Bioscience). Mice were killed if they became morbid or had a weight loss of 20% or greater, and survival was determined. In the second model, 2 × 10^7^ K562 cells (mixed 1:1 with matrigel) were injected subcutaneously into the lower right flank of C.B17 SCID mice (Taconic Laboratories, Hudson, NY, USA). After tumors were established (11 days later), mice were randomized into two groups (eight mice/group) matched for tumor volume. Mice were then orally dosed with vehicle or PTC596 (20 mg/kg) once a week, and tumor sizes were measured. In the third model, 1 × 10^7^ HL-60 cells were injected intravenously into NOD-SCID mice. Three days after injection, mice were randomized into three groups. Mice were orally dosed with vehicle (9 mice) or PTC596 (10 or 12.5 mg/kg, seven mice/group) twice a week until death, and survival was determined. To investigate if PTC596 affects normal hematopoiesis, the spleen colony-forming unit assay was performed. Bone marrow cells (50 000 cells) were collected from donor C57BL/6 mice (three mice/group) 2 weeks after a single oral dose of vehicle, two oral doses of 20 mg/kg PTC596 given 1 week apart or a single intraperitoneal injection of 150 mg/kg 5-FU. The bone marrow cells (5 × 10^4^ cells/mouse) were transplanted into lethally irradiated (10 Gy) mice (10 mice/group). Eleven days after transplantation, macroscopic colonies were counted after fixation of spleens in Carnoy's solution.

### Statistical analysis

Statistical analysis was carried out using the two-tailed Student *t*-test, Mann–Whitney *U*-test, paired *t*-test or one-way analysis of variance with Dunnett's *post hoc* multiple comparison test. *P*-values<0.05 were considered statistically significant. Unless otherwise indicated, average values are expressed as the mean±s.d. Power calculations were used to determine the appropriate sample sizes in mice experiments. The Kaplan–Meier method was used to generate survival curves for animal studies, and the log-rank test was used to test the survival difference among the groups of subjects with different treatments.

## Results

### PTC596 inhibits cell growth and induces apoptosis in AML cell lines in a p53-independent manner

**Figure 1:**
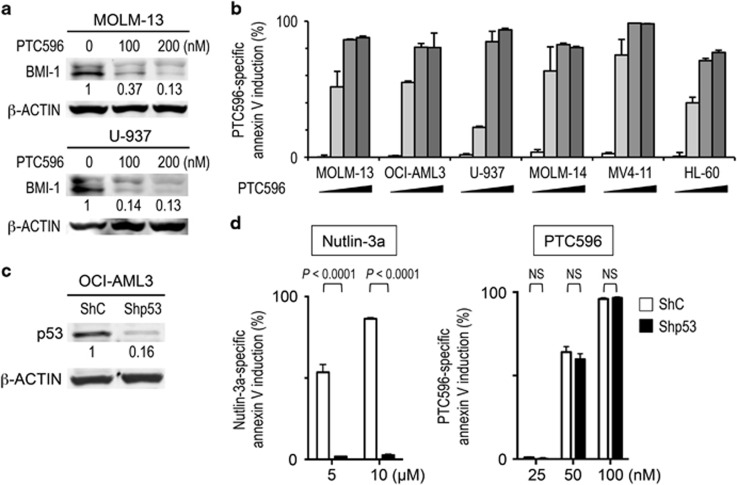
The novel BMI-1 inhibitor PTC596 induces apoptosis in AML cells in a p53-independent manner. (**a**) Expression of BMI-1 protein in MOLM-13 and U-937 cells treated for 20 h with various concentrations of PTC596 as indicated. The intensities of immunoblot signals were quantified and normalized to those of β-ACTIN. Levels in untreated cells were set at 1.0. (**b**) AML cell lines were incubated with 20, 50, 100 or 200 nM PTC596 for 48 h, and annexin V-positive fractions were measured by flow cytometry. (**c**) p53 expression was determined by western blot analysis in OCI-AML3 cells, which were transduced with retroviruses encoding either scrambled shRNA (ShC) or p53-specific shRNA (Shp53). Intensity of the immunoblot signals was quantified and the relative intensity compared to β-Actin was calculated. (**d**) Wild-type p53 OCI-AML3 cells transduced with lentivirus encoding either ShC or Shp53 were incubated with the indicated concentrations of Nutlin-3a (wild-type p53 inducer) or PTC596 for 72 h, and annexin V-positive fractions were determined. The results are expressed as the mean±s.d. of triplicate measurements. NS, not significant.

After we confirmed that BMI-1 expression is reduced in AML cells after exposure to PTC596 (Figure 1a), we examined the effect of PTC596 on the viability of AML cell lines. The ED_50_ (effective concentration inducing 50% cell killing as measured by annexin V positivity) and IC_50_ (concentration at which cell growth is inhibited by 50%) values at 48 h are summarized in Table 1. PTC596-induced apoptosis, as evidenced by annexin V positivity, was directly correlated with the percentage of trypan blue uptake (data not shown). PTC596 induced apoptosis in a dose- (Figure 1b) and time-dependent (Supplementary Figure S1a) manner. The average IC_50_ and ED_50_ values among six cell lines were 30.7±4.1 nM and 60.3±6.7 nM (mean±s.e.m.), respectively, indicating nanomolar potency. Importantly, p53 mutant U-937 and HL-60 cells were as sensitive to PTC596 as p53 wild-type cells (Figure 1b and Table 1). The basal level of BMI-1 was not correlated with IC_50_ (*r*=0.019, *P*=0.97) or ED_50_ (*r*=0.21, *P*=0.68; Table 1).

To determine whether PTC596 induces p53-independent apoptosis, we examined the effect of PTC596 on stable p53 knockdown OCI-AML3 cells. Western blot analysis revealed that p53-specific short hairpin RNA reduced p53 levels by 84% (Figure 1c). The selective MDM2 inhibitor Nutlin-3a has been found to potently reactivate wild-type p53 and induce apoptosis in AML.^32^ p53 knockdown cells were significantly less sensitive to Nutlin-3a compared with control cells expressing a scrambled target sequence, indicating an efficient inhibition of p53-induced apoptosis (Figure 1d). In contrast, PTC596 induced apoptosis both in p53 knockdown OCI-AML3 cells and control cells (Figure 1d). Stable p53 knockdown cells were also generated in MOLM-13 and MV4-11 cells, and p53-specific short hairpin RNA reduced p53 levels by 69% and 90%, respectively. As shown in Table 1, the IC_50_ and ED_50_ values for PTC596 were not significantly different between p53 knockdown and control cells.

We next investigated if PTC596 treatment induced p53 in AML cells, as BMI-1 may potentially suppress p53 through ARF–MDM2-p53 signaling. The MDM2 inhibitor Nutlin-3a served as positive control. As shown in Supplementary Figure S1b, PTC596 did not increase p53 levels in any of the three AML cell lines examined. To investigate the biological consequences of BMI-1 expression in AML, BMI-1 levels were reduced by siRNA transfection in MOLM-13 cells. BMI-1-specific siRNA led to reduced BMI-1 protein expression by 51% but p53 expression level was not increased in BMI-1 knockdown cells (Supplementary Figure S1c). These data suggest that p53-mediated signaling is not involved in PTC596-induced apoptosis.

### BMI-1 overexpression desensitizes AML cells to PTC596-induced apoptosis

**Figure 2:**
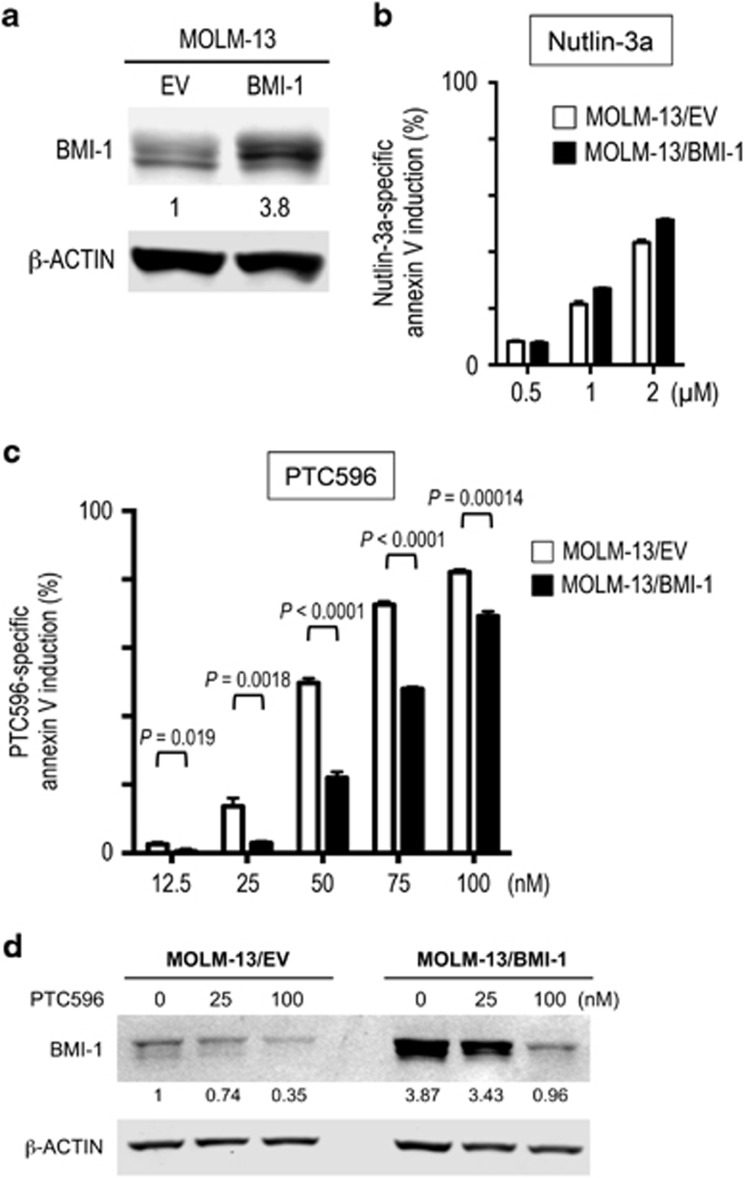
BMI-1 overexpression desensitizes AML cells to PTC596-induced apoptosis. (**a**) Expression of BMI-1 protein in MOLM-13 cells overexpressing BMI-1 (MOLM-13/BMI-1) or empty vector control (MOLM-13/EV). The intensities of immunoblot signals were quantified and normalized to β-ACTIN. Levels in MOLM-13/EV cells were set at 1.0. (**b**, **c**) MOLM-13/BMI-1 and MOLM-13/EV cells were incubated with the indicated concentrations of Nutlin-3a (**b**) or PTC596 (**c**) for 48 h, and annexin V-positive fractions were measured by flow cytometry. The results are expressed as the mean±s.d. of triplicate measurements. (**d**) MOLM-13/BMI-1 and MOLM-13/EV cells were incubated with the indicated concentrations of PTC596 for 48 h, and expression of BMI-1 protein was determined. The intensities of immunoblot signals were quantified and normalized to those of β-ACTIN. Levels in untreated MOLM-13/EV cells were set at 1.0.

We next investigated if overexpressed BMI-1 protects leukemia cells from PTC596-induced apoptosis. MOLM-13 cells overexpressing BMI-1 (MOLM-13/BMI-1) showed 3.8 times higher levels of BMI-1 than empty vector control (MOLM-13/EV) cells (Figure 2a). Although BMI-1 levels did not affect cell sensitivity to Nutlin-3a (Figure 2b), MOLM-13/BMI-1 cells were significantly less sensitive to PTC596 than MOLM-13/EV cells (Figure 2c). Higher concentration of PTC596 reduced BMI-1 expression levels even in MOLM-13/BMI-1 cells (Figure 2d), which might be associated with the smaller protective effect. We also established a stable K562 cell line with overexpressed BMI-1, and again, BMI-1 overexpression desensitized cells to PTC596-induced apoptosis (Supplementary Figure S2). These data indicate on-target BMI-1 inhibition by PTC596.

### PTC596 causes mitotic arrest in a p53-independent manner

**Figure 3:**
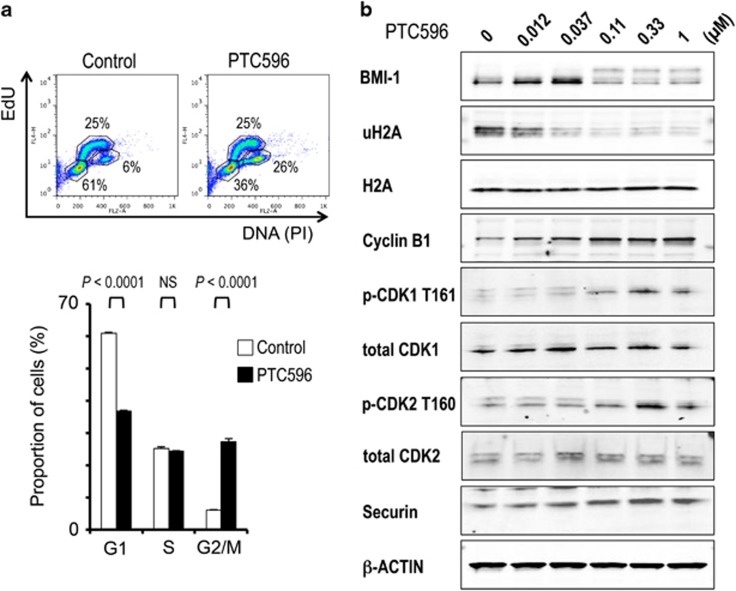
PTC596 causes mitotic arrest in AML cells. (**a**) MOLM-13 cells were exposed to 200 nM PTC596 for 10 h and cell cycle progression was determined. The dot plot represents EdU incorporation (*y* axis) versus DNA content, as determined by PI staining (*x* axis). The results are expressed as the mean±s.d. of triplicate measurements. NS, not significant. (**b**) Expression of BMI-1, ubiquitinated histone H2A (uH2A), H2A and G2/M cell cycle regulator proteins in MOLM-13 cells treated with the indicated concentrations of PTC596 for 20 h.

Because PTC596 showed potent anti-proliferative effects against AML cells independent of p53 mutation status, we investigated the cell cycle distribution of p53 wild-type MOLM-13 and p53-defective U-937 cells after a 10-h treatment with 200 nM PTC596. At this time point, PTC596 did not show significant apoptosis. PTC596 treatment led to an accumulation of cells in G2/M phase, whereas the percentage of cells in G1 phase decreased both in MOLM-13 (Figure 3a) and U-937 cells (Supplementary Figure S3a), indicating p53-independent G2/M arrest. May-Grünwald–Giemsa staining showed morphological features of mitotic arrest, including breakdown of the nuclear membrane and chromatin condensation in MOLM-13 and U-937 cells after 10 h of exposure to 200 nM PTC596 (Supplementary Figure S3b).

To further clarify PTC596-induced cell cycle arrest in AML, changes in protein levels of G2/M regulators were determined in MOLM-13 cells after exposure to PTC596. As shown in Figure 3b, treatment with PTC596 at 110 nM or higher concentrations significantly reduced protein levels of BMI-1 and its downstream target ubiquitinated histone H2A. In accordance with previous studies,^28^ a shifted band of BMI-1, which has been thought to be phosphorylated BMI-1, was observed along with a decrease in non-phosphorylated BMI-1 levels. Levels of phosphorylated CDK1 (T161) and phosphorylated CDK2 (T160) were both increased, supporting the observed accumulation of cells in G2/M phase. More specifically, PTC596 increased cyclin B1 and securin levels, indicating that the cells were not able to progress beyond the spindle checkpoint.

### PTC596 reduces anti-apoptotic MCL-1 levels and induces mitochondrial apoptosis in AML

**Figure 4:**
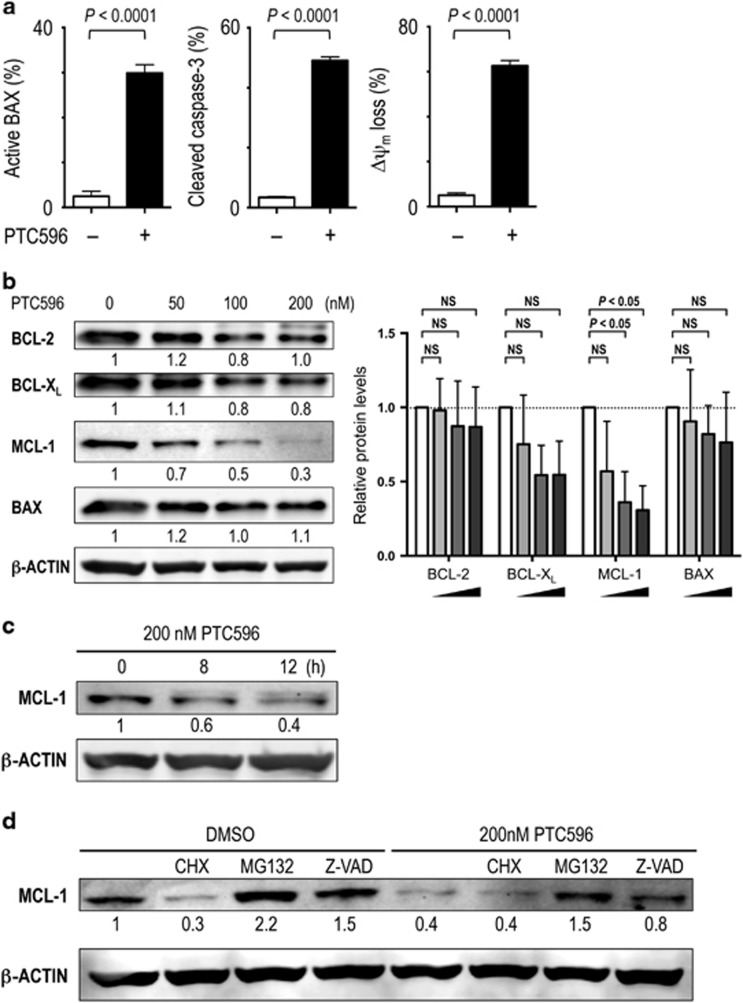
PTC596 reduces anti-apoptotic MCL-1 levels and induces mitochondrial apoptosis in AML. (**a**) BAX conformational changes, caspase-3 cleavage and Δψ_m_ loss were determined by flow cytometry in MOLM-13 cells after 16-h (BAX activation) or 20 h (caspase-3 cleavage and Δψ_m_ loss) exposure to 200 nM PTC596. The results are expressed as the mean±s.d. (**b**) Expression of BCL-2 family proteins in MOLM-13 cells after 20-h treatment with 50, 100 or 200 nM PTC596. The intensities of immunoblot signals were quantified and normalized to those of β-ACTIN. Levels in untreated cells were set at 1.0. White bars represent untreated controls. One-way ANOVA with Dunnett's test was used to determine significance between control and treated samples. NS, not significant. (**c**) MCL-1 expression in MOLM-13 cells treated with 200 nM PTC596 for indicated time periods. (**d**) MOLM-13 cells were preincubated for 1 h with 7 μM cycloheximide (CHX), 0.5 μM MG132 or 25 μM Z-VAD-FMK, and MCL-1 levels were determined after 20-h treatment with 200 nM PTC596. β-ACTIN was used as loading control. Results are representative of three independent experiments.

To investigate if PTC596 induces apoptosis through the intrinsic pathway, BAX conformational change, caspase-3 cleavage and ΔΨ_m_ were determined in MOLM-13 and U-937 cells. PTC596 induced BAX activation, caspase-3 cleavage and ΔΨ_m_ loss in both cell lines (Figure 4a and Supplementary Figure S4a), indicating mitochondrial apoptosis. As BCL-2 family proteins maintain ΔΨ_m_, BCL-2, BCL-X_L_, MCL-1 and BAX protein levels were determined. Interestingly, PTC596 decreased MCL-1 levels in a dose-dependent manner in both cells (Figure 4b and Supplementary Figure S4b), indicating a p53-independent effect. Levels of BCL-2, BCL-X_L_ and BAX were not changed significantly. The reduction of MCL-1 started as early as 8 h after exposure (Figure 4c). BMI-1 levels were reduced in MOLM-13 and U-937 cells using siRNA. As shown in Supplementary Figure S5a, acute *BMI1* knockdown was associated with decreased levels of MCL-1. Neither PTC596 treatment nor siRNA-induced *BMI1* knockdown affected expression levels of *MCL1* mRNA (Supplementary Figure S5b).

To elucidate how BMI-1 inhibition by PTC596 reduces MCL-1 levels, we treated MOLM-13 cells with 200 nM PTC596 for 20 h, either alone or in combination after 1-h preincubation with 7 μM cycloheximide, 0.5 μM MG132 or 25 μM Z-VAD-FMK. The protein synthesis inhibitor cycloheximide did not significantly affect the rate of decrease in MCL-1 levels after PTC596 treatment (Figure 4d), implying that PTC596 inhibits new protein synthesis. The proteasome inhibitor MG132 and the pan-caspase inhibitor Z-VAD-FMK both blocked MCL-1 reduction after PTC596 treatment (Figure 4d), suggesting that PTC596 enhances proteasome- and caspase-mediated degradation of MCL-1. These data suggest that PTC596 reduces MCL-1 expression through post-transcriptional protein synthesis inhibition and accelerated degradation, destabilizing mitochondrial membrane potential and inducing mitochondrial apoptosis.

### PTC596 induces apoptosis in CD34^+^CD38^low/−^ AML stem/progenitor cells

**Figure 5:**
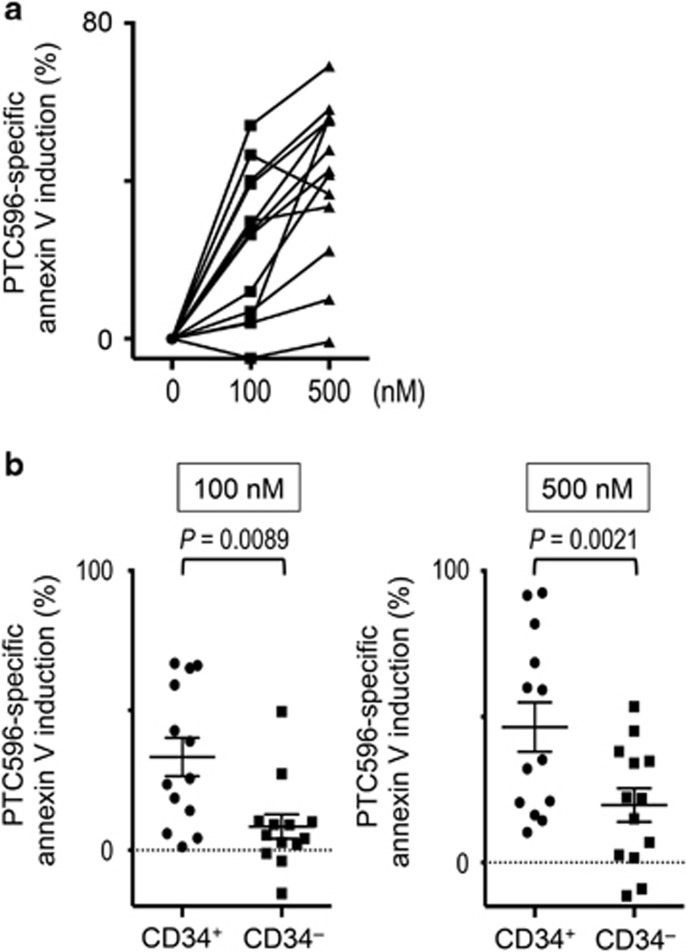
PTC596 induces apoptosis in primary AML samples, particularly in primitive CD34^+^ blasts. (**a**) Thirteen primary AML cells were incubated with the indicated concentrations of PTC596 for 72 h and annexin V-positive fractions were measured by flow cytometry. (**b**) PTC596 sensitivity of the primitive CD34^+^ subpopulation was compared with that of the more mature CD34^–^ subpopulation. One sample was excluded from the analysis owing to the absence of CD34+ cells. Error bars indicate s.e.m.

We next examined the apoptotic effects of PTC596 on primary cells from 13 patients with AML. The patient characteristics are summarized in Table 2. Five patients harbored a complex karyotype that is closely associated with mutant p53 status. As shown in Figure 5a, treatment of primary AML cells with PTC596 caused a dose-dependent increase in the percentage of annexin V-positive cells. A PTC596-specific increase in the proportion of annexin V-positive cells of >30% was observed in 10 of 13 (77%) primary AML samples after exposure to 500 nM PTC596. Importantly, immature CD34^+^ (CD38^low/−^) AML cells were more susceptible to PTC596 than mature AML cells (Figure 5b, Supplementary Figure S6a). In contrast, complex karyotype was not a predictor for an inferior sensitivity to PTC596 (Supplementary Figure S6b). Two PTC596-sensitive samples with complex karyotype were available for p53 sequence analysis. One sample had a non-functional p53 mutation (p.M237K), implying that PTC596 induces apoptosis in p53-defective cells (Supplementary Figure S6).

To investigate if PTC596 reduces MCL-1 expression in AML stem/progenitor cells, cellular MCL-1 levels were determined in an AML patient sample using CyTOF. As shown in Supplementary Figure S7, PTC596 reduced MCL-1 expression in AML cells, which was most prominent in the CD34^+^CD38^low/−^ stem/progenitor population. MCL-1 expression is controlled by multiple signaling pathways.^33, 34^ We therefore investigated the upstream activators and signaling proteins for MCL-1, including p-ERK1/2 (Thr202/Tyr204), p-AKT (Ser473), p-STAT3 (Thy705) and p-STAT5 (Thy694). In accordance with changes in MCL-1, PTC596 reduced protein expression of MCL-1 inducers, which was most prominent with p-AKT (42.8% reduction). The PI3K/Akt pathway has been shown to promote protein synthesis of MCL-1 while inhibiting its degradation.^35, 36^

### PTC596 inhibits myeloid leukemia cell growth *in vivo* while sparing normal hematopoietic cells

**Figure 6:**
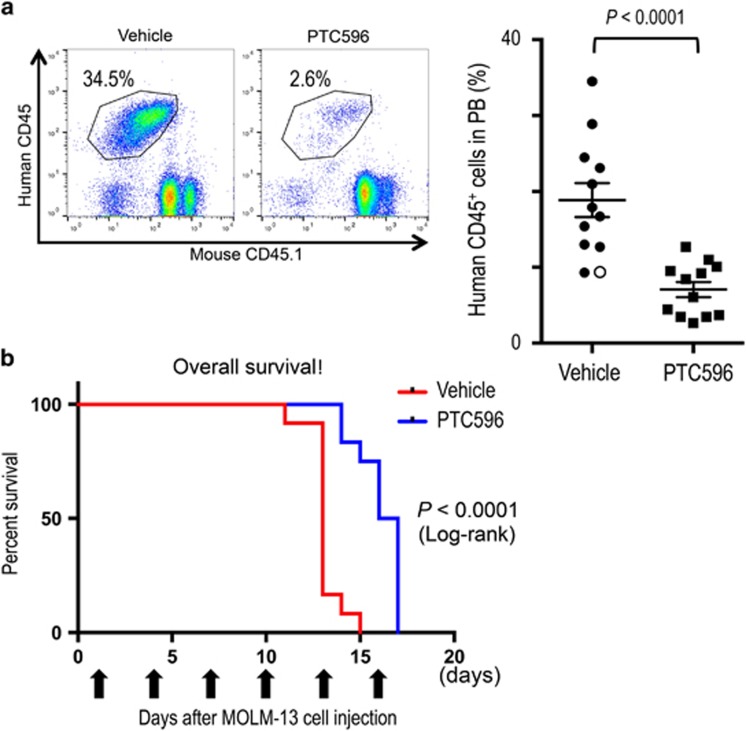
PTC596 attenuates AML growth *in vivo*. (**a**) NSG mice were injected intravenously with MOLM-13 cells and were randomized into PTC596- and vehicle-treated groups. Leukemia burden expressed as percent human CD45^+^ cells is shown in peripheral blood (PB) on day 13 after treatment initiation. In one vehicle-treated mouse, PB was collected on day 11 as it became moribund (open circle). (**b**) Survival of MOLM-13 xenograft mice after treatment with PTC596 or vehicle control. Survival comparison was made with log-rank test.

We investigated *in vivo* anti-leukemia activities of PTC596 using myeloid leukemia xenograft models. In a highly aggressive MOLM-13 xenograft model, there was a significant reduction of circulating human CD45-positive cells after 13 days of treatment (Figure 6a). Peripheral blood was collected on day 11 from a vehicle-treated mouse as it became moribund, and circulating leukemia cells were detected in the mouse (open circle; Figure 6a). Survival of PTC596-treated mice was significantly longer than the vehicle-treated group (median survival: 16.5 days versus 13.0 days, *P*<0.0001 by log-rank test; Figure 6b). In the K562-bearing mice, the mean tumor volume of mice treated with PTC596 was significantly smaller than that of control mice (Supplementary Figure S8a). Anti-leukemic activity of PTC596 was further confirmed in the HL-60 xenograft model, in which PTC596 significantly prolonged mouse survival compared with the vehicle-treated mice in a dose-dependent manner (Supplementary Figure S8b). The effect of PTC596 on normal hematopoiesis was determined by the spleen colony-forming unit assay, and the colony numbers were not statistically different between the PTC596- and vehicle-treated groups (Supplementary Figure S8c).

## Discussion

In this study, we provide evidence that the small molecule BMI-1 inhibitor PTC596, currently in phase 1 clinical trial in patients with advanced solid tumors, induces mitochondrial apoptosis in AML cell lines and primary blasts. The major advantages of this novel strategy to target BMI-1 in AML by PTC596 were as follows: (1) nanomolar potency; (2) high apoptotic activity against CD34^+^CD38^low/−^ stem/progenitor cells in primary blasts; and (3) p53 independency in inducing apoptosis. In AML, p53 mutations have been almost exclusively found in patients with unfavorable complex karyotype or therapy-related AML.^4, 5^ In addition, p53 mutation is itself an independent predictor of extremely poor outcome.^6^ Therefore, PTC596 deserves further evaluation in clinical trials for refractory or relapsed AML, regardless of the presence of unfavorable karyotype or p53 mutations in leukemia blasts.

BMI-1 resides upstream of ARF in the BMI-1–ARF–MDM2–p53 signaling pathway and may potentially promote MDM2-mediated p53 degradation.^7^ In addition, BMI-1 has been recently found to directly bind to p53 and promote p53 ubiquitination and degradation.^37^ One may therefore expect that BMI-1 inhibition would lead to increased levels of cellular p53. In AML cells, however, neither PTC596 treatment nor acute BMI-1 knockdown by siRNA increased p53 protein levels. Previous studies have shown that conventional *Bmi1* knockout (*Bmi1*^−/−^) mice develop bone marrow failure and die <2 months after birth.^10, 38, 39, 40^ However, p53 expression was not determined in those studies. Primary mouse embryonic fibroblasts derived from *Bmi1*^−/−^ embryos and those from *Bmi1*^+/+^ embryos express similar protein levels of p53.^41^ Higher p53 expression in *Bmi1* gene-deleted cells compared with *Bmi1*^+/+^ cells were reported in neuronal cells, but the differences were modest (less than twofold) at the protein level.^42, 43^ p53 expression has been investigated in association with *BMI1* overexpression or knockdown. In breast cancer cells, modulation of *BMI1* did not significantly change p53 protein levels.^44^ In contrast, BMI-1 has been shown to have a negative impact on p53 expression in neuroblastoma cell lines.^37, 45^ Calao *et al.*^37^ showed that BMI-1 promotes p53 ubiquitination and degradation, and Cui *et al.*^45^ demonstrated that BMI-1 blocks MYCN-driven p53 induction. We speculate that BMI-1 regulates p53 under specific circumstances or in specific cell types. A p53-independent activity of PTC596 might support the idea that p53-mediated signaling is dispensable for apoptosis induction by BMI-1 inhibition in AML.

We found that PTC596 treatment downregulates MCL-1 and induces mitochondrial apoptosis in AML cells. Some researchers have observed that BMI-1 positively regulates MCL-1 expression,^27, 46^ although the mechanism was not formally identified. MCL-1 is a critical anti-apoptotic BCL-2 family protein in AML.^17, 18, 47, 48, 49, 50^ MCL-1 is highly expressed in AML cells,^17, 18, 47, 48^ especially in the CD34^+^CD38^−^ stem/progenitor cell population.^47, 48^ Recently, MCL-1 has been reported to be essential for AML development, survival and drug resistance.^49, 50^ In addition, elevated expression of MCL-1 at the time of relapse was reported in AML patients.^51^ MCL-1 is acutely regulated with a half-life of ~30 min, and its expression is controlled by multiple signaling pathways, including STAT3/5, PI3K/AKT and MEK/ERK pathways.^33, 34, 35, 36^ Among these pathways, the PI3K/AKT pathway has been shown to be upregulated by BMI-1,^52, 53^ and in turn upregulates MCL-1 by stimulating protein synthesis and inhibiting protein degradation.^35, 36^ In our study, PTC596 affected multiple processes of MCL-1 protein synthesis and degradation while reducing phosphorylated AKT levels, which may support the hypothesis that BMI-1 inhibition by PTC596 downregulates MCL-1 through PI3K/AKT pathway inhibition. The inhibitory effect of the BMI-1 inhibitor PTC596 on MCL-1 expression may enhance its therapeutic activity against AML stem/progenitor cells.

As BMI-1 is expressed in normal hematopoietic stem cells, the development of hematologic toxicity might be a concern in patients treated with PTC596. Recently, interim results of the first-in-human phase 1 study of PTC596 in advanced solid tumors have been reported.^54^ In the study, 19 patients have been enrolled at doses of 0.65, 1.3, 2.6, 5.2 and 10 mg/kg PTC596 and the plasma concentrations increased in a dose-proportional manner. Plasma concentrations at doses ⩾2.6 mg/kg exceeded those demonstrated to be efficacious in mouse xenograft models. Interestingly, hematologic toxicity equal to or greater than grade 2 has been observed only in one patient who received 10 mg/kg PTC596 and developed grade 4 neutropenia and thrombocytopenia. The ongoing clinical study would clarify if PTC596 has an acceptable toxicity profile in patients.

In summary, our data indicate that the novel BMI-1 inhibitor PTC596 downregulates MCL-1 and induces mitochondrial apoptosis in a p53-independent manner. PTC596 effectively killed CD34^+^CD38^−^ AML stem/progenitor cells while sparing normal hematopoietic stem/progenitor cells. Our findings strongly encourage the development of BMI-1-targeted therapy for AML patients, especially for those with unfavorable complex karyotype or therapy-related AML that are frequently associated with p53 mutations.

## Figures and Tables

**Table 1:** ED_50_, IC_50_, p53 mutational status and BMI-1 protein levels in AML cell lines

*Cell lines*	*ED_50_ (n**M**)*	*IC_50_ (n**M**)*	*p53 mutation status*	*BMI-1 levels (MFIR)*
MOLM-13	61.5	22.4	Wild-type	3.7
OCI-AML3	60.3	45.6	Wild-type	1.0
MOLM-14	52.5	22.4	Wild-type	4.7
MV4-11	41.6	40.6	Wild-type	1.1
U-937	55.4	27.9	Mutant	5.3
HL-60	90.4	25.1	Mutant	0.7
OCI-AML3 ShC	52.5	30.9		
OCI-AML3 Shp53	57.2	29.7		
MOLM-13 ShC	32.8	20.5		
MOLM-13 Shp53	44.7	21.4		
MV4-11 ShC	57.5	41.1		
MV4-11 Shp53	66.3	34.5		

Abbreviations: MFIR, mean fluorescence intensity ratio; ShC, shRNA control. Shp53, p53 shRNA. MFIR was normalized to that of OCI-AML3 cells.

**Table 2:** Clinical data for AML patients

*No.*	*Age (y/sex)*	*Status*	*Cytogenetics*	*Source*	*Blasts (%)*	*CD34*^a^ *(%)*
1	71/F	New	46,XX	PB	> 90	98.9
2	76/M	New	Complex	PB	28.8	92.3
3	52/M	New	46,XY	BM	79.2	72.2
4	54/M	New	46,XX,t(8;21)(q22;q22)	BM	78.4	82.9
5	58/M	New	46,XY	BM	> 90	1.3
6	68/M	New	Complex	PB	34	NA
7	69/M	New	Complex	PB	40	NA
8	38/F	New	46,XX	BM	84.8	2.9
9	66/M	New	46,XY,del(7q)	BM	26	53.0
10	84/F	New	Complex	PB	60	NA
11	86/M	New	Complex	BM	48	87.0
12	58/M	Relapse	46,XY	PB	84	0.2
13	87/F	Relapse	46,XX,inv(16)(p13.1;q22),add(18)(q21)	BM	60	7.0

Abbreviations: BM, bone marrow; NA, not analyzed; PB, peripheral blood.

a The percentage of CD34^+^ cells was determined by flow cytometry in isolated bone marrow mononuclear cells or peripheral blood mononuclear cells.
