# Supplementary figures and images for: The novel BMI-1 inhibitor PTC596 downregulates MCL-1 and induces p53-independent mitochondrial apoptosis in acute myeloid leukemia progenitor cells
Source: Blood Cancer J. 2017 Feb 17;7(2):e527–. doi: 10.1038/bcj.2017.8 (PMC5386342; doi:10.1038/bcj.2017.8)

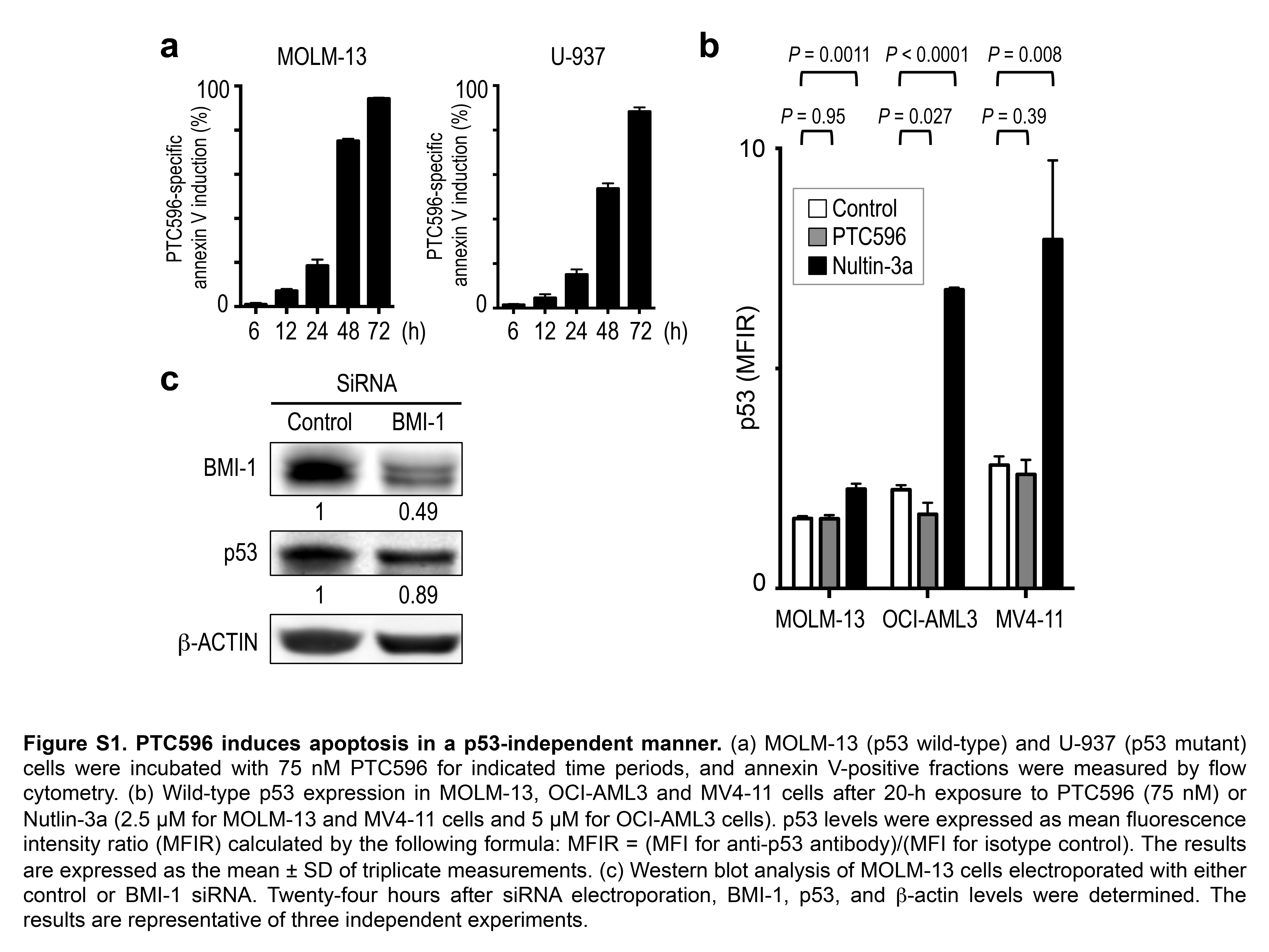


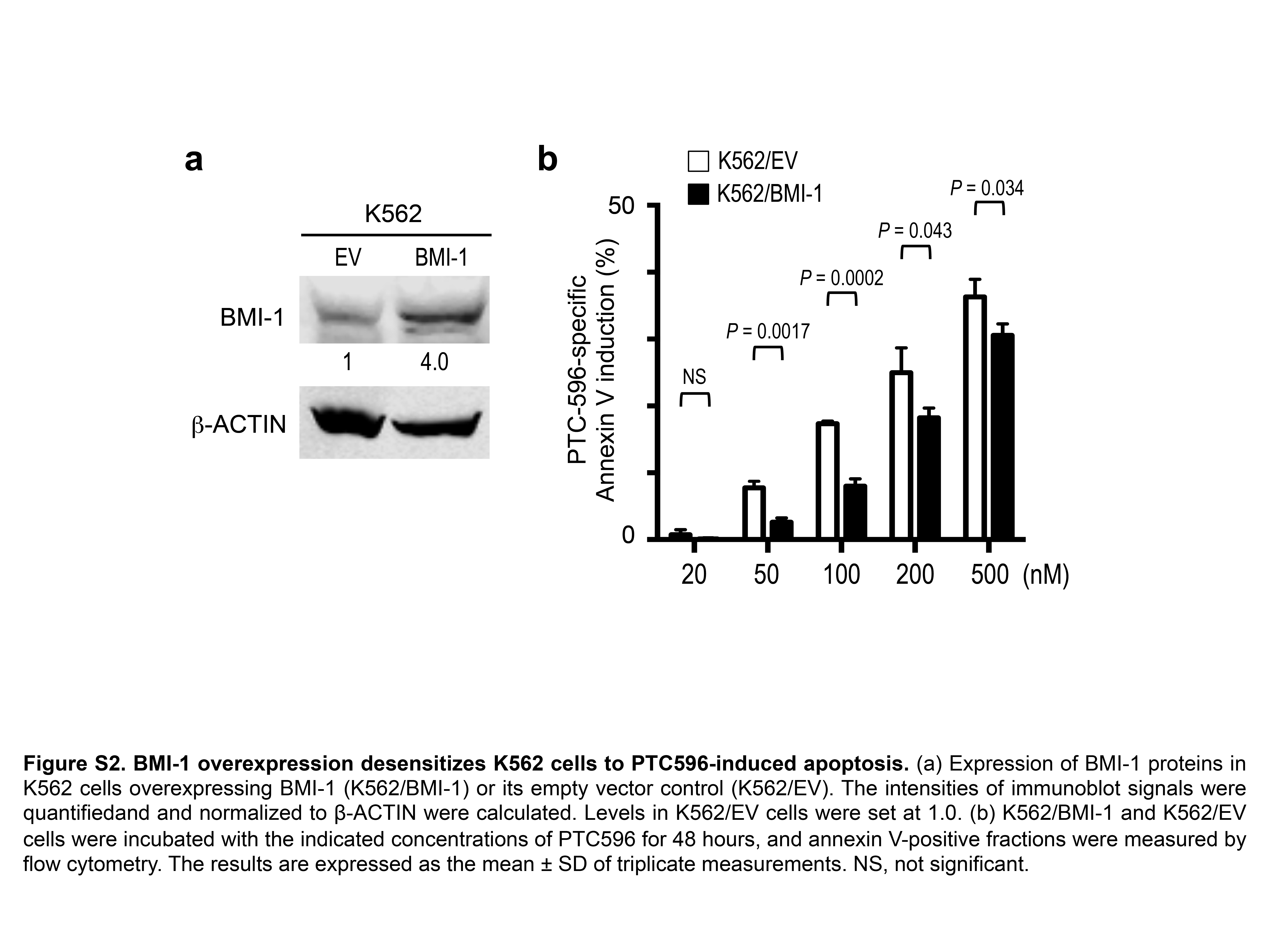


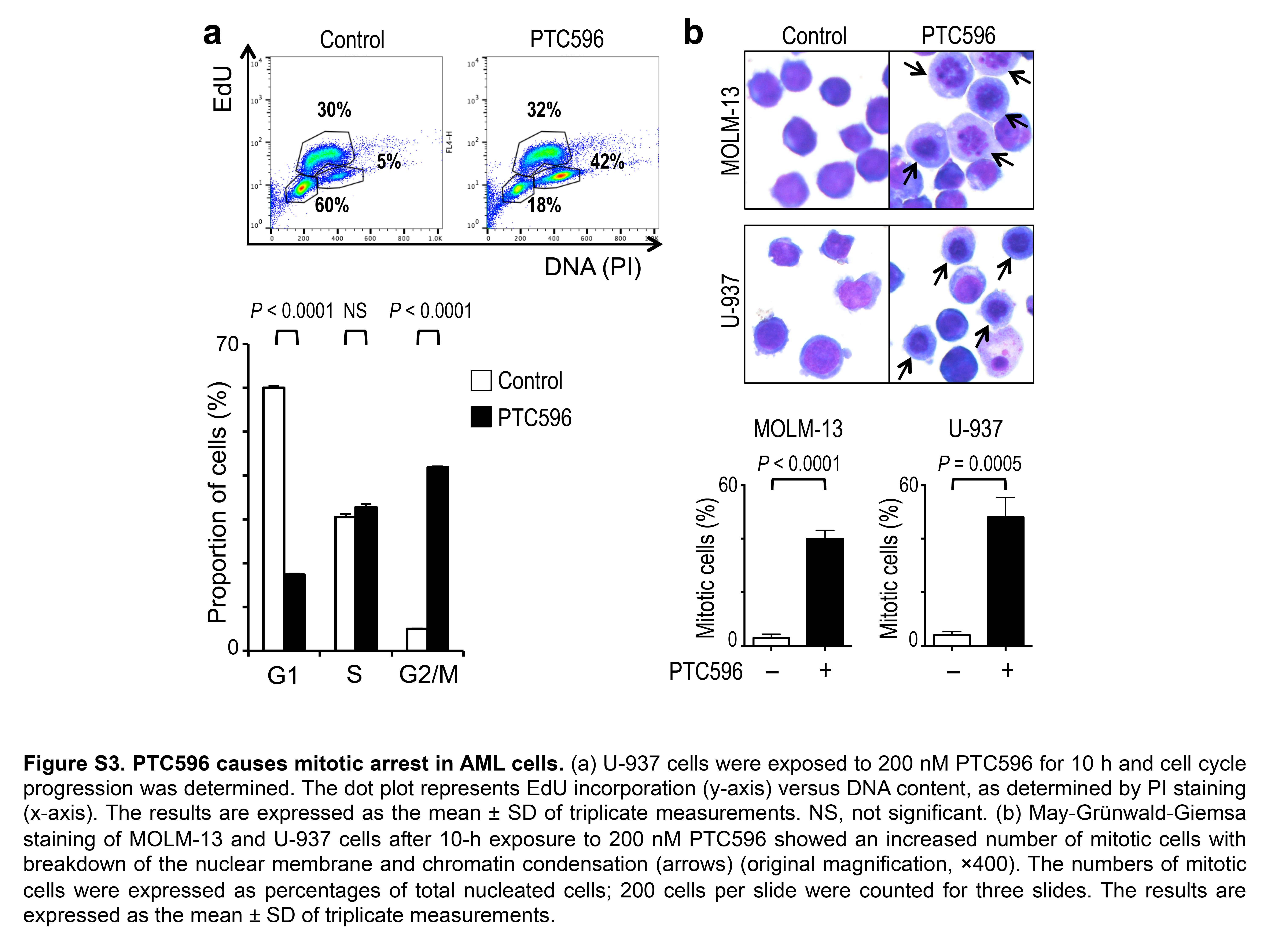


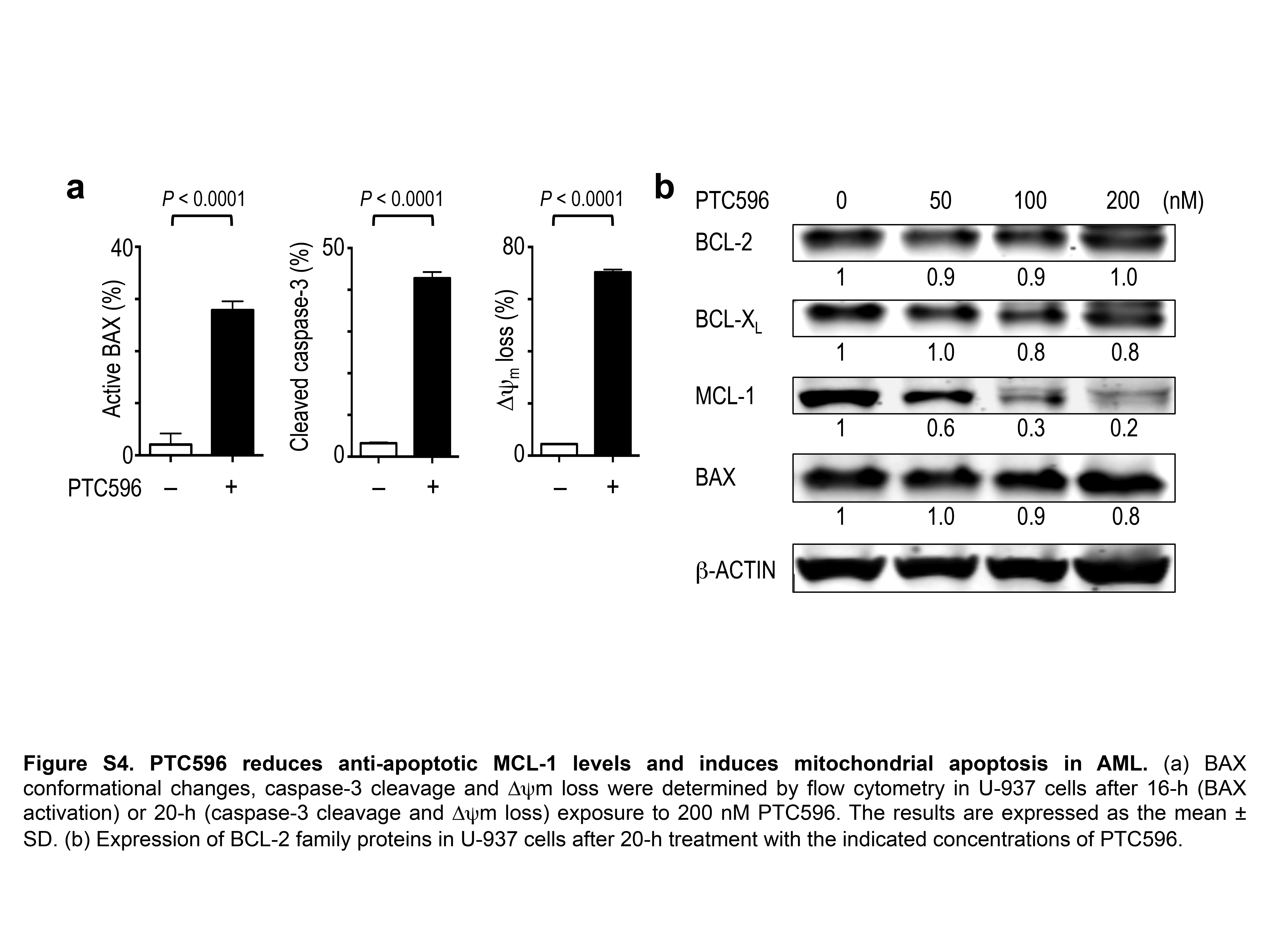


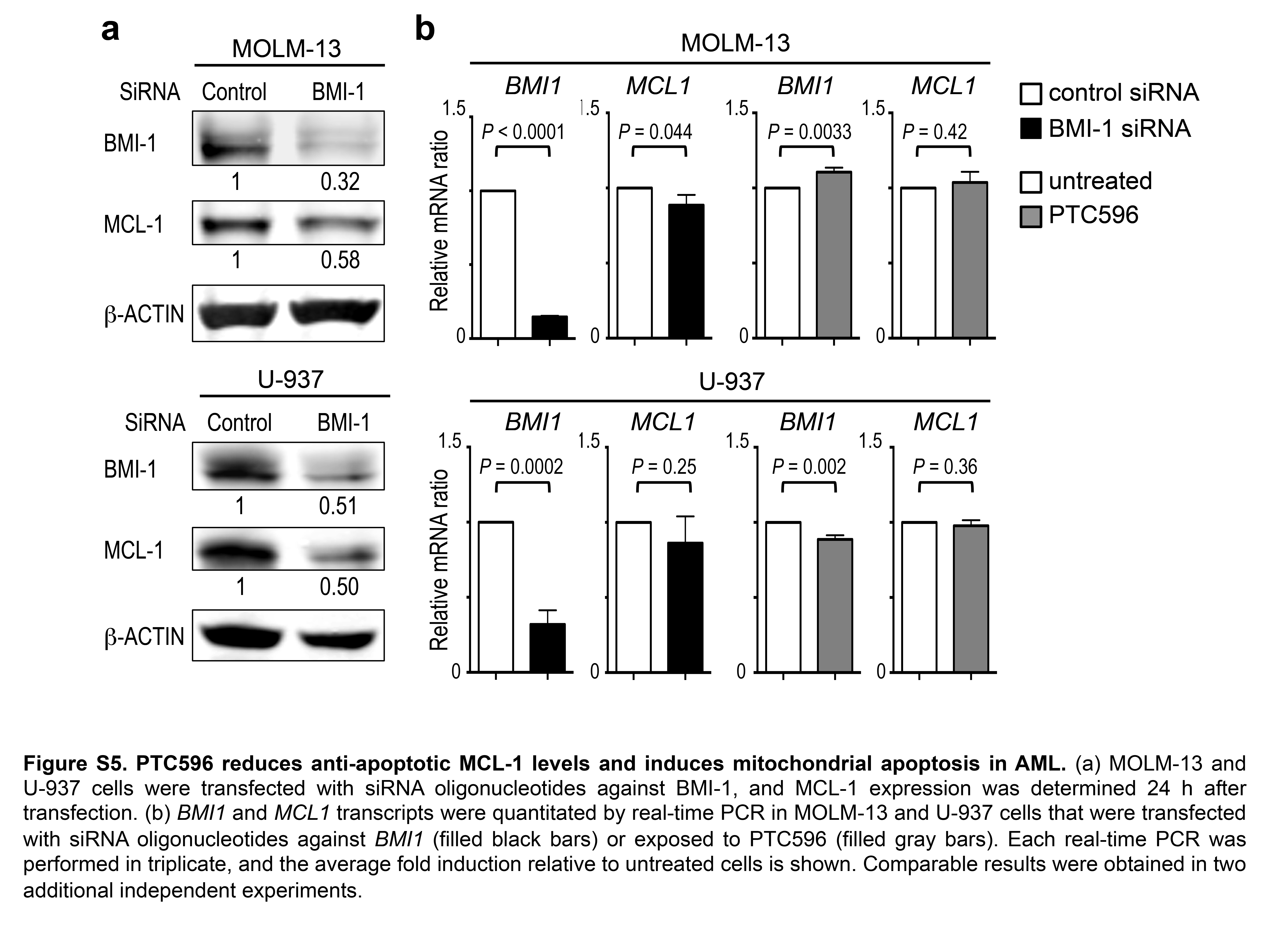


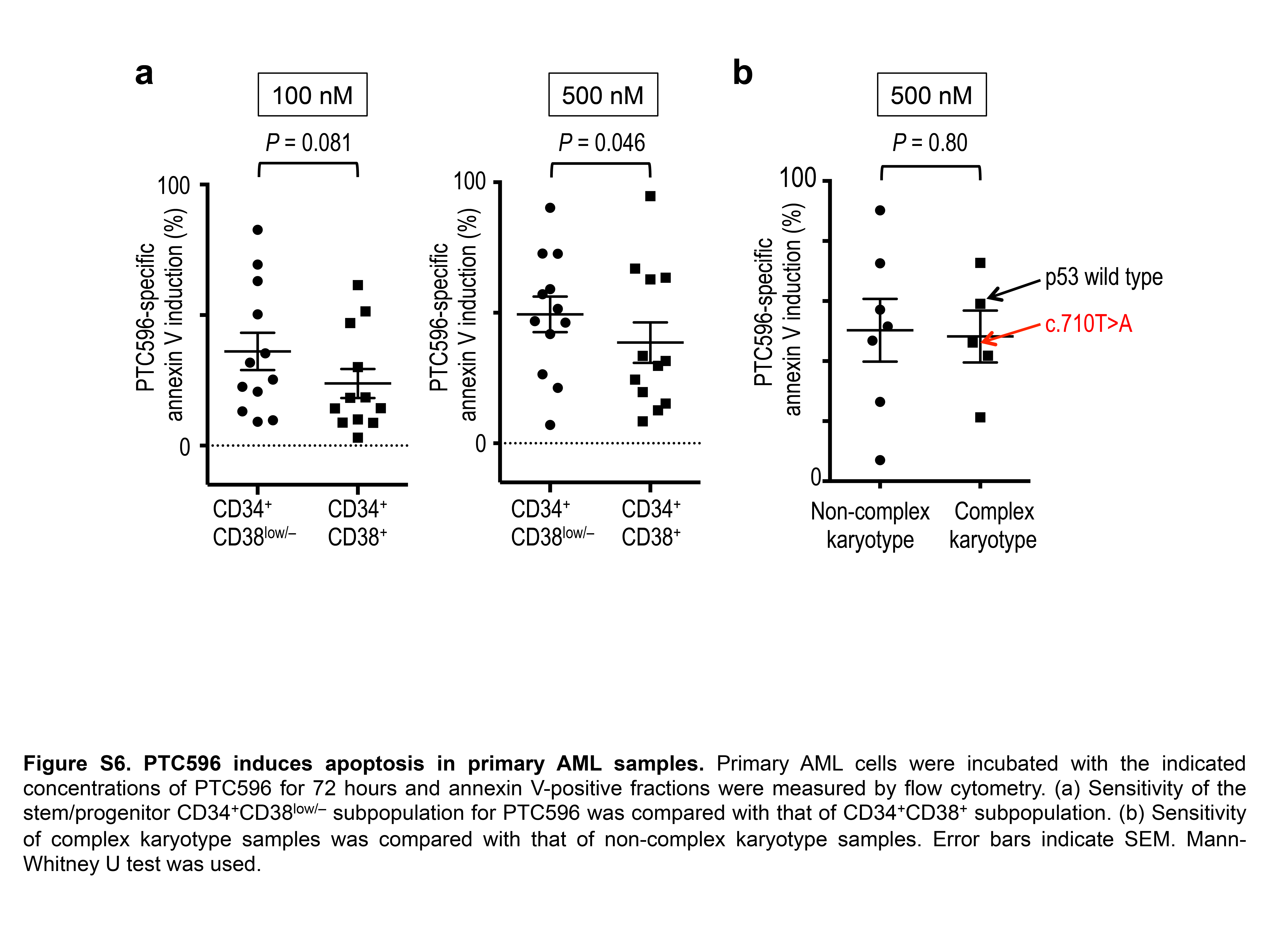


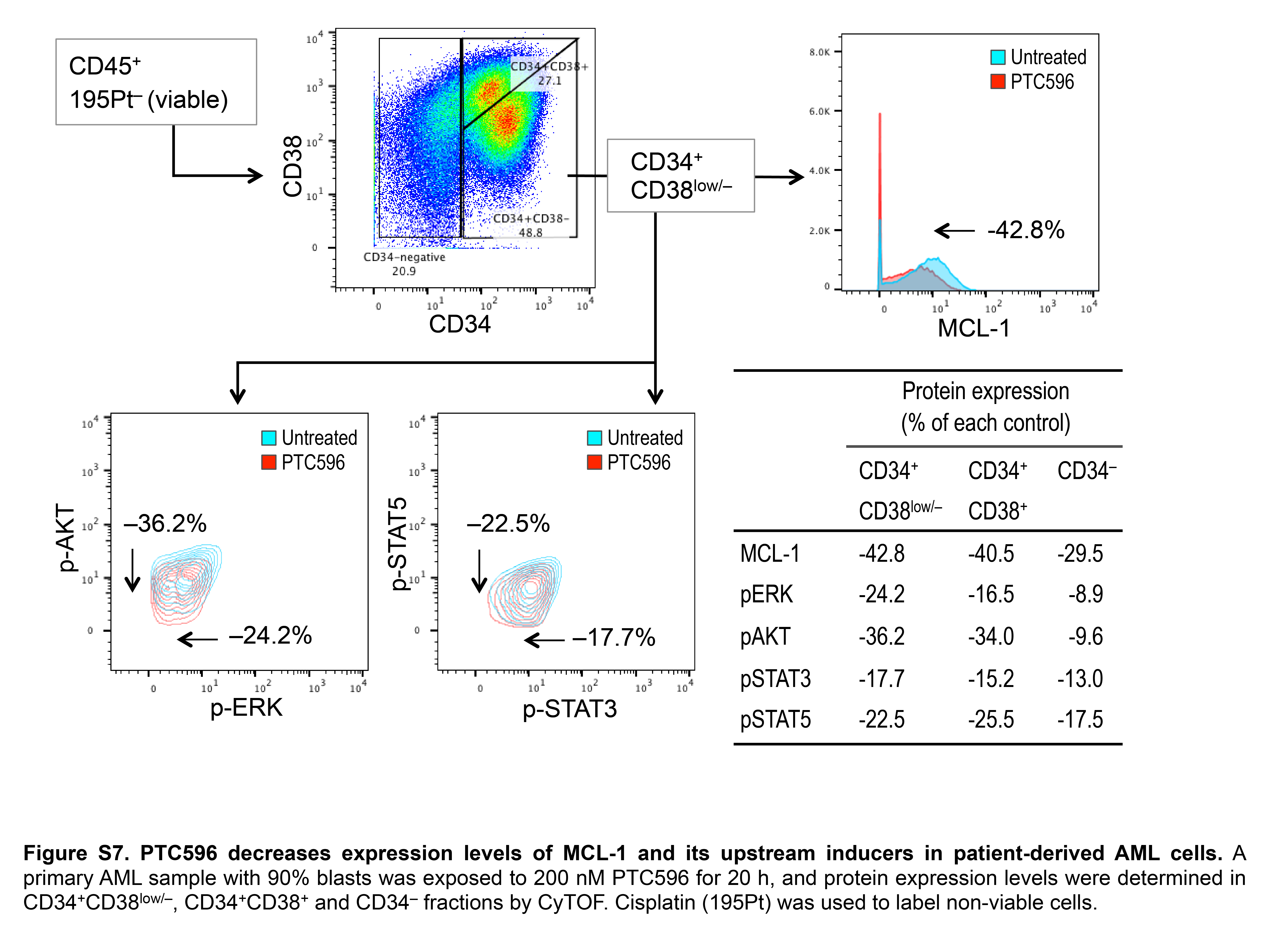


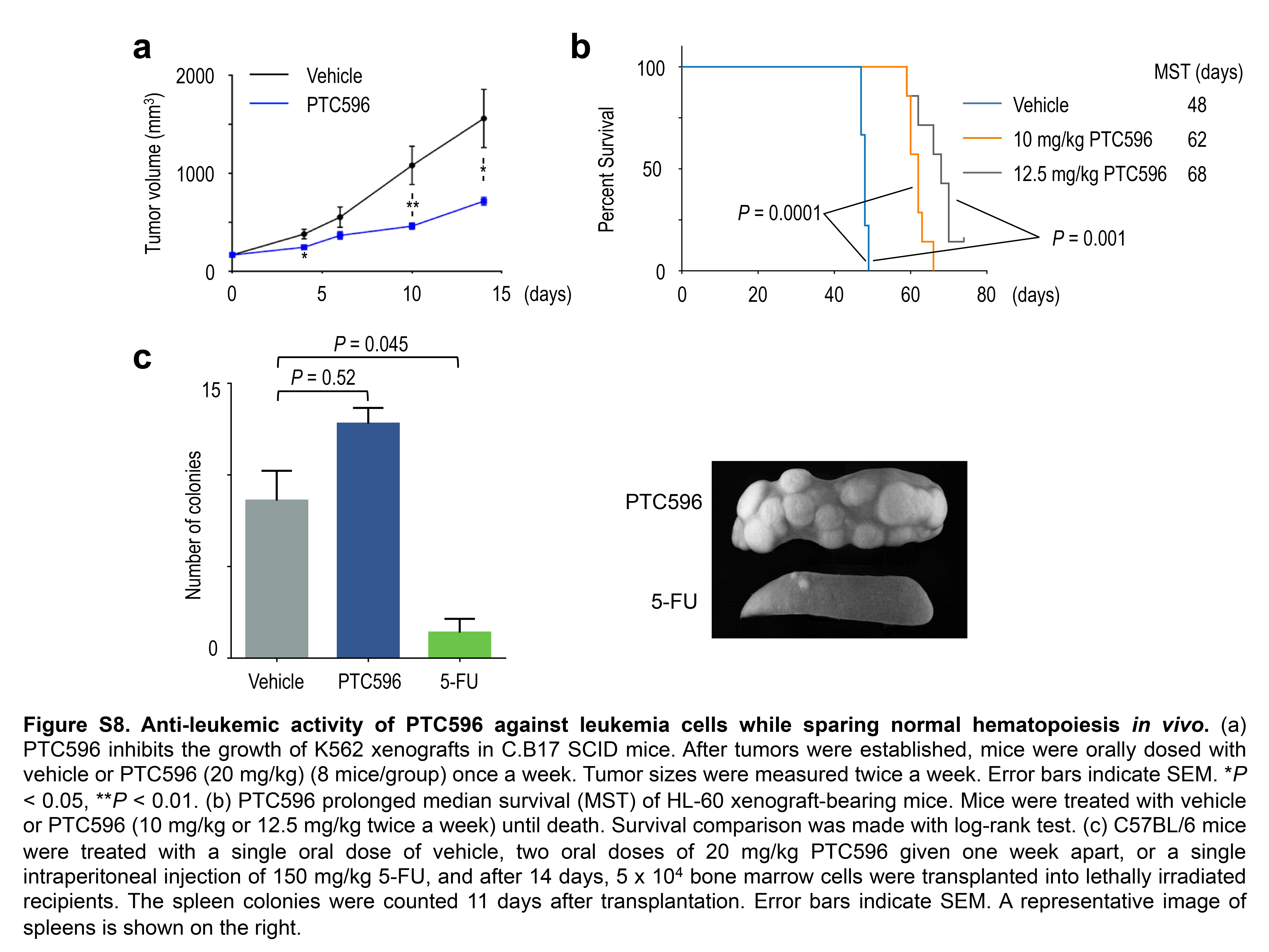

Supplement: Supplementary Information [file bcj20178x1.docx]
